# Portuguese Physical Literacy Assessment for adolescents (15–18 years): validation using confirmatory factor and composite analyses

**DOI:** 10.3389/fspor.2023.1192025

**Published:** 2023-06-27

**Authors:** João Mota, João Martins, Marcos Onofre, Dean Dudley

**Affiliations:** ^1^ Sports Studies and Physical Education Programme, School of Education, College of Arts, Celtic Studies & Social Sciences, University College Cork, Cork, Ireland; ^2^ Centro de Estudos em Educação, Faculdade de Motricidade Humana, Universidade de Lisboa, Estrada da Costa, Oeiras, Portugal; ^3^ UIDEF, Instituto de Educação, Universidade de Lisboa, Alameda da Universidade, Lisbon, Portugal; ^4^ Macquarie School of Education, Macquarie University, Sydney, NSW, Australia; ^5^ School of Human Movement and Nutrition Sciences, University of Queensland, Brisbane, QLD, Australia; ^6^ Centre of Educational Measurement and Assessment, University of Sydney, Sydney, NSW, Australia

**Keywords:** physical education, construct validity, reliability, high school, physical literacy, confirmatory factor analysis, confirmatory composite analysis, adolescents

## Abstract

**Objective:**

The aim of this study was to assess the construct validity and reliability of the Portuguese Physical Literacy Assessment (PPLA) instruments (a questionnaire and a tool using teacher-reported data). We also investigated the conceptual and practical implications of reflective vs. formative measurement of Physical Literacy using the PPLA.

**Methods:**

Multiple Confirmatory Factor Analysis (CFA) and Confirmatory Composite Analysis (CCA) models were used complementarily to assess construct validity in a sample of 521 grade 10–12 Portuguese students from Lisbon, Portugal. Bifactor model-based indices (*ω*), Explained Common Variance (ECV), and Percentage of Uncontaminated Correlations (PUC) were used to assess score reliability and adequacy.

**Results:**

Using CFA, an asymmetrical bifactor model (S*1-1) provided the best fit to the data [Robust Comparative Fit Index = 97, Robust Root Mean Square Error Of Approximation = 0.05 (0.04–0.06), Standardized Root Mean Square Residual (SRMR) = 0.04], while CCA resulted in the best absolute fit for single first-order composite models (*d*_G_, *d*_L_, and SRMR below or borderline of their 95% critical value). Through a reflective paradigm, the total PL score should not be used in isolation (ECV = 0.49, *ω*_H_ = 0.71, lower than recommended 0.80). Subscales for the Physical, Psychological, and Social domains attained acceptable reliability scores (*ω*_s_ = 0.76, 0.82, 0.80, and 0.60).

**Conclusions:**

A general trait of PL accounts for considerable variance in all indicators. We advise calculation of a total summed PL score and domain scores, which should be interpreted conjointly in applied settings. Despite both paradigms being tenable, future research efforts should use a bifactor measurement model, which permits disentanglement of the variance attributed to the general PL trait and its domains. Overall, evidence supported the construct validity and reliability of the PPLA for its intended use as an integrated tool to measure PL as a multidimensional construct in 15- to 18-year-old Portuguese students in a physical education setting.

## Introduction

Physical literacy (PL) is a holistic concept referring to the skills and attributes that individuals demonstrate through physical activity (PA) and movement throughout their lives, enabling them to lead healthy and fulfilling lifestyles (1). This multidimensional concept is argued as the foundation for many international physical education (PE), sport, and public health agendas (2–5). In recent years, multiple efforts have been made by diverse authors and research groups based around the globe (e.g., Australia, Canada, China, France, and Iran) to develop and refine measurement instruments that assess an individual's physical literacy journey (6–13).

The Portuguese Physical Literacy Assessment (PPLA) was developed as a tool composed of two instruments (a questionnaire, PPLA-Q, and an observational instrument, PPLA-O) to be used in PE to provide a feasible and holistic assessment of adolescent's PL in grades 10–12 (15–18 years) of high school. It was inspired by the Australian Physical Literacy Framework (APLF) (14), which is a conceptual model of PL learning composed of 30 elements across four learning domains (Physical, Psychological, Social, and Cognitive). The PPLA was also informed by the outcomes and didactic philosophy of the Portuguese PE syllabus (15, 16). Previous studies using Item Response Theory models have supported construct validity and reliability of both the PPLA-Q and PPLA-O at the elemental level (17–19). However, the higher-order dimensionality of these tools requires further investigation.

Assessment of their dimensionality can be assessed through structural equation modeling (SEM) whereby two main approaches are undertaken depending on the auxiliary theories assumed to underlie measurement (20–22). In this study, they were reflective measurement and formative measurement, whereas previous studies on PL measurement have always implicitly assumed a multidimensional reflective view, modeling PL as a (a) correlated factor (7, 13, 23) or (b) higher-order factor (10, 24). However, there are ontological and conceptual issues inherent to both approaches, which are reviewed in Supplementary Material S1.

Our initial model for the PPLA (25) hypothesized PL as a higher-order composite formed of domain-specific composites, based on the idea of non-exchangeability of domains and indicators, along with the assertion that variation in each of the domains would be plausibly independent of each other (e.g., one could conceive that an increase in cognitive-related skills would not be simultaneous with an increase in psychological-related attitudes). Despite this, given the recency of PL construct testing, it is cogent to test alternative competing models that could further provide practical and conceptual advantages.

As such, this study sought to establish evidence supporting construct validity and reliability of the PPLA by integrating measures derived from the PPLA-Q and PPLA-O. It then compared results drawn from factor-based (reflective) methods and composite-based (formative) methods. Based on these findings, it then assessed the adequacy of using a PL total score and respective subscales. As a secondary research aim, we investigated the implications of the different methods for the wider conceptual understanding of PL.

## Materials and methods

### Participants

**Figure 1:**
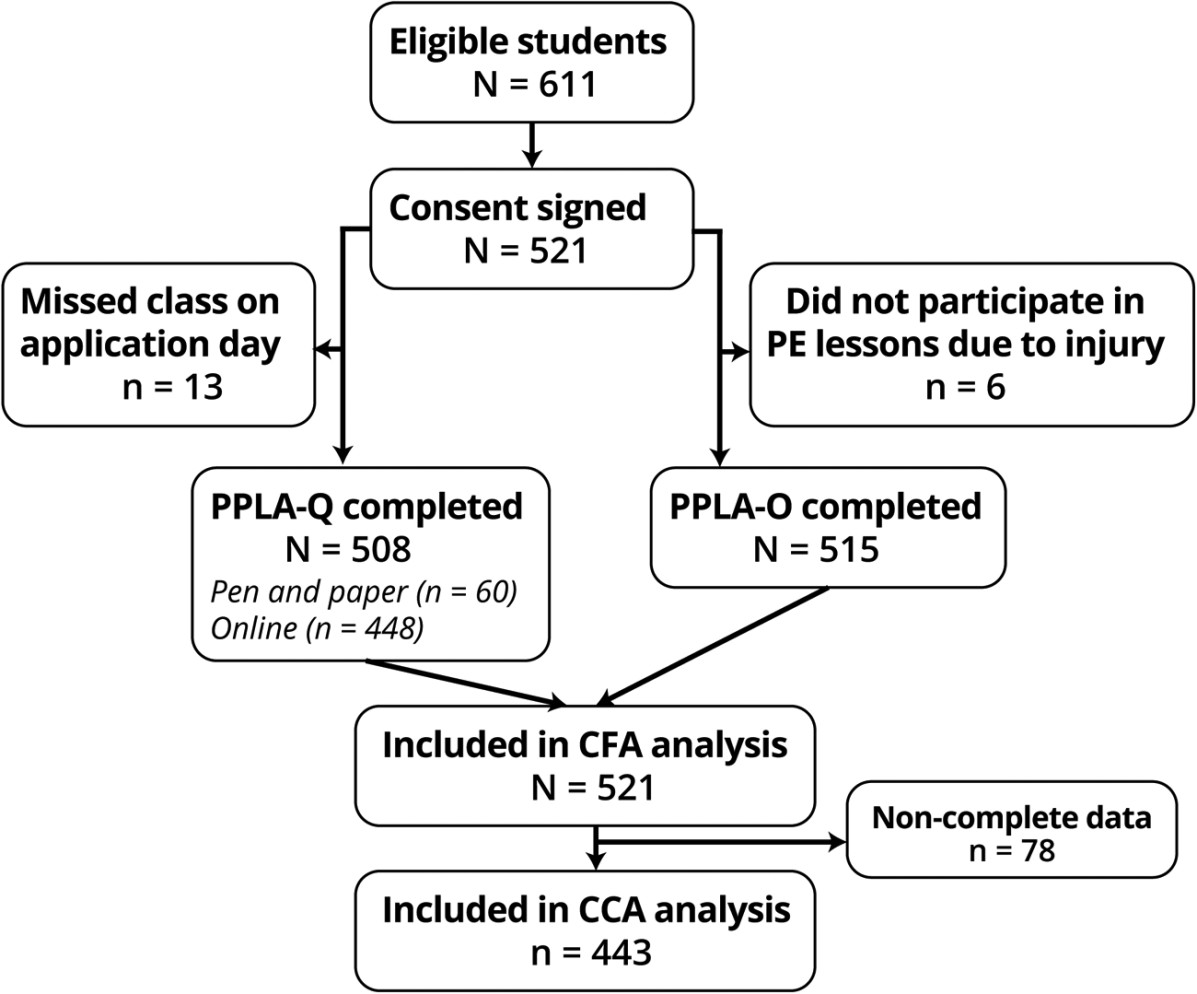
Portuguese physical literacy assessment validation participant flowchart. CFA, confirmatory factor analysis; CCA, confirmatory composite analysis.

A sample of 521 (58% female) grade 10–12 students (*M*_age_ = 16, SD = 1 years) from six public schools in Lisbon's metropolitan area was used (25 classes, 22 different PE teachers). Sampling procedures and full sample characteristics are detailed in prior work (17, 18). Briefly, recruitment was stratified by grade and course major according to population percentage quotas. Schools from diverse socioeconomic backgrounds were chosen to increase sample representativeness. The participant recruitment and participation flow are shown in Figure 1.

A minimum sample size of 275 was initially chosen based on a power analysis conducted in *WarpPLS* software (26), using the Inverse Square Root Method (27), for a minimum absolute path coefficient of 0.15 and power of 0.80. Data collection occurred between January and March 2021. PPLA-Q was self-administered (students) both in a paper and online format in the presence of the lead investigator, and PPLA-O was self-administered (PE teachers) using an online spreadsheet.

### Measures

#### PPLA measures

##### Physical domain

The Physical domain of the PPLA was assessed through the PPLA-O (18). It is composed of two modules: *Movement Competence, Rules, and Tactics (MCRT)* and *Health-related fitness (HRF)*. The MCRT includes two scores—Manipulative-based activities and Stability-based activities—and these were calculated through a two-factor Graded Response Model (GRM; an Item Response Theory model). These scores summarize the general movement competence (including tactical decision and rule knowledge) of the student in physical activities, which elicit mostly manipulative movement skills (e.g., team-sports), and which elicit mostly stability movement skills (e.g., gymnastics). To facilitate interpretation, factor scores derived with Expected *A Posteriori* (EAP) scoring were transformed into a 0–100 score.

Health-related physical fitness module included seven indicators, all assessed through existing FITescola® protocols (28), in three major subareas: (1) *Cardiorespiratory endurance* was assessed through the 20-m Progressive Aerobic Cardiorespiratory Endurance Run (PACER), using the number of laps completed; (2) *Muscular endurance* was assessed through the number of executions in the Curl-Up and the 90° Push-Ups protocols; (3) *Flexibility* was assessed through the Backsaver Sit-and-Reach (lower body) measured in centimeters for each leg and Shoulder Stretch (upper body)—with binary coding (unable/able) for each arm—protocols. All these protocols are routinely applied by PE teachers and are part of teachers’ initial education curriculum.

##### Psychological domain

The Psychological domain included four indicators assessed through the PPLA-Q: *Motivation*, *Confidence*, *Emotional Regulation*, and *Physical Regulation*. All these indicators comprised the total summed score of responses in each respective scale (composed of seven, nine, seven, and eight items, respectively), and then transformed into a percentage of maximum points (0–100 score) to normalize the different number of items in each scale. All scales have been calibrated through Mokken Scale Analysis [e.g., (29)] using nonparametric Item Response Theory (IRT) models, and have shown evidence supporting good score reliability (Molenaar–Sijtsma's ***ρ*** of 0.83 to 0.94) (30) and construct validity in this sample (17): dimensionality (Loevinger's *H* of .47 to .66) (31), discriminant validity (deattenuated correlations between subscales of 0.27 to 0.73) and convergent validity.

##### Social domain

The Social domain included four indicators: *Culture*, *Ethics*, *Collaboration,* and *Relationships*. These indicators followed the same logic as those of the Psychological domain presented above, using a total summed score across the seven and six items (for the last three mentioned subscales), respectively. Previous validation using Mokken Scale Analysis (17) resulted in good score reliabilities (***ρ*** of 0.86 to .0.91) and construct validity (*H* of 0.54 to 0.64; deattenuated correlations of 0.18 to 0.74).

##### Cognitive domain

The Cognitive domain was assessed through a single indicator: *Content Knowledge.* Its score was derived from calibration of an IRT model (mixed 2-parameters nested logit and graded response model) on 10 response items dealing with knowledge in 5 main content themes (19). This calibration gathered evidence on construct validity and score reliability of the test (marginal reliability of 0.60) to distinguish students with descriptive (*Foundation*) knowledge from those with higher analytical knowledge (*Mastery*). Factor scores derived with EAP were transformed into a 0–100 score.

#### Self-reported physical activity

The short form of the *International Physical Activity Questionnaire* (IPAQ-SF) (32) was used to obtain weighted estimates of each intensity of physical activity per week (MET/min/week). No total summed score was used since this instrument has shown different validity across intensities (33, 34) and since it is tenable that different intensities might interact differently with the different domains of PL.

### Statistical analysis

All statistical analyses used RStudio 1.4.1106 (35), with *R* 4.0.1 (36). Missing data and descriptive statistics (Table 1) were computed using the packages *naniar* (37) and *psych* (38). A statistic of *χ*^2^ (593) = 791.65, *p* < 0.001 with 38 missing patterns, in Little's test (39) provided evidence against data Missing Completely at Random. Missing data most likely originated from students missing class on the day of measures’ application and therefore is tenable to assume a Missing at Random (MAR) mechanism occurred.

**Table 1:** Descriptive statistics for measures in the PPLA-questionnaire and PPLA-observation (*N* = 521).

Variable	*n* missing (%)	M (SD)	Median	Univariate normality	
				Shapiro–Wilk *W*	*p*-Value
Self-reported PA					
Vigorous^a^	22 (4.2)	2,071 (2,084.2)	1,440.0	0.85	<0.001
Moderate^a^	26 (5.0)	1,071 (1,122.2)	720.0	0.80	<0.001
Walking^a^	26 (5.0)	767.5 (950.1)	396.0	0.76	<0.001
PPLA-O measures					
PACER	22 (4.2)	49.5 (22)	44.0	0.93	<0.001
Push-ups	26 (5.0)	18.1 (9.6)	18.0	0.93	<0.001
Curl-ups	23 (4.4)	48.6 (21.7)	45.0	0.91	<0.001
Shoulder stretch (frequency of achievement)					
Right	83 (15.9)	95%		0.21	<0.001
Left	83 (15.9)	89%		0.37	<0.001
Sit and reach (cm)					
Right	85 (16.3)	30.7 (8.3)	31.0	0.99	0.009
Left	84 (16.1)	30.2 (8.2)	31.0	0.99	0.006
Manipulative-based activities^b^	6 (1.2)	54.9 (21.8)	55.4	0.99	0.076
Stability-based activities^b^	6 (1.2)	43.4 (16.4)	43.7	0.98	<0.001
PPLA-Q measures					
Content knowledge^b^	13 (2.5)	68.8 (15.2)	70.0	0.99	<0.001
Motivation^b^		74.9 (14.8)	77.1	0.97	<0.001
Confidence^b^		68.4 (16.8)	68.9	0.98	<0.001
Emotional regulation^b^		69.9 (14.6)	71.4	0.98	<0.001
Physical regulation^b^		75.1 (12.2)	75.0	0.98	<0.001
Culture^b^		58.7 (19.5)	57.1	0.98	<0.001
Ethics^b^		81.5 (11.9)	83.3	0.92	<0.001
Collaboration^b^		85.0 (11.4)	86.7	0.94	<0.001
Relationships^b^		77.7 (13.5)	80.0	0.97	<0.001

PACER, progressive aerobic cardiovascular endurance run; PA, physical activity; PPLA-O, PPLA-observation; PPLA-Q, PPLA-questionnaire.

^a^ MET/week.

^b^ Maximum score = 100.

Data were screened for univariate and multivariate normality through the *MVN* package (40); however, the shoulder stretch assessment had to be removed from the latter test to achieve convergence due to it being a binary indicator. Results of the univariate tests are presented in Table 1. Mardia's statistics (skewness = 2,739.39, *p* < 0.001; kurtosis = 13.33, *p *< 0.001) render any normality assumption untenable.

Data were screened for multivariate outliers using the Minimum Covariance Determinant approach (41) through the *Routliers* package (42) that highlighted 69 multivariate outliers. Sensitivity analysis revealed no differences in model fit or parameters in the main analysis and so outliers were kept in the analyses. Bivariate Pearson and polyserial correlations between measures were obtained in *polycor* (43) and reported in Table 3. For factor-based analysis, the *Push-ups* indicator was multiplied by a factor of 2 to rescale its variance.

#### Confirmatory factor analysis

**Figure 2:**
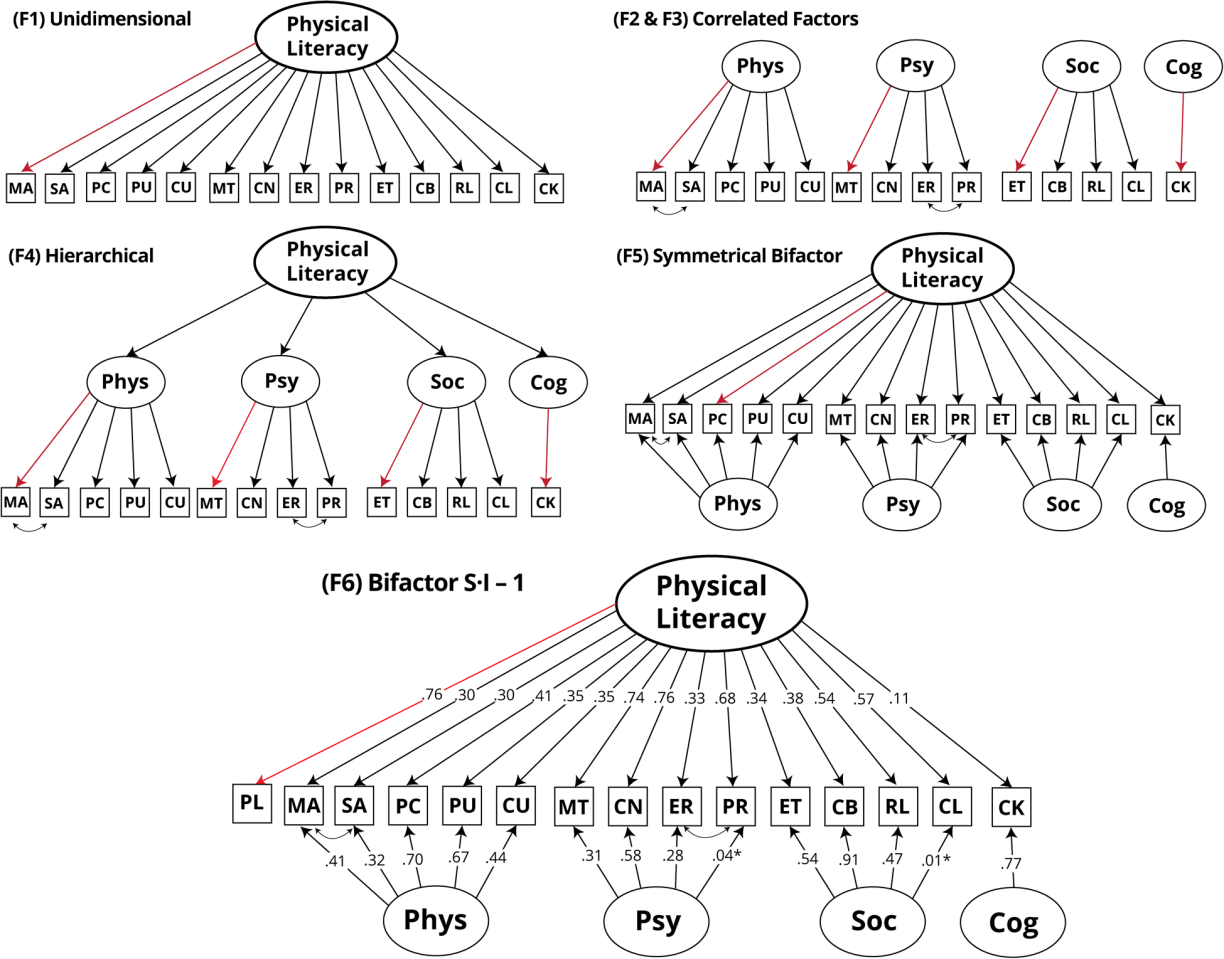
Factor-based models estimated in the study, standardized factor loadings are presented in F6. PL, physical literacy; MA, manipulative-based activities; SA, stability-based activities; PC, PACER; PU, push-ups; CU, curl-ups; MT, motivation; CN, confidence; ER, emotional regulation; PR, physical regulation; ET, ethics; CB, collaboration; RL, relationships; CL, culture; CK, content knowledge. Marker indicators are colored red; error terms are omitted for clarity; F2 is equal to F3 without freely estimated covariances between indicators.

Since PPLA is based on the APLF (44), a clear rationale for factorial structure has been previously defended (25). We employed CFA to test the previously hypothesized model structure against other tenable competing models presented in the literature (10, 24) by adding the assumption of a reflective measurement model. Six models were estimated (see Figure 2), which included unidimensional, correlated first-order factors, second-order, canonical (symmetric) bifactor, and bifactor S·I-1 models. All models were estimated in *lavaan* 0.6.9 (45) with the included variables being specified as continuous. Given the violation of multivariate normality, robust maximum likelihood estimation (MLR) with robust “Huber-White” standard errors (46) and a scaled test statistic (equivalent to Yuan-Bentler T2*) (47) were also used. Based on the existence of missing data on various variables and the assumption of MAR, Full Information Maximum likelihood (FIML) (48) was used to estimate unbiased parameters (49).

In all models, the metric of latent factors was fixed by using the first indicator as a marker. Error covariances were constrained to zero unless otherwise specified. In all multiple factors models, the Cognitive domain factor was specified as a single indicator, and its error variance was constrained to (1 − reliability) × variance × (indicator) (50, 51). Estimation of the last three models (F4-F6) used bounded parameters to stabilize the solution (52). Initial estimation of model F5 resulted in a negative variance (Heywood case); however, changing the marker indicator resolved this issue. For the sixth model, a five-point global indicator of PL (“*I can lead a healthy and active life*”) was inserted into the model. A sensitivity analysis compared MLR estimation with weighted least square mean and variance adjusted (WLSMV) estimation, with no substantial differences in fit indices or parameters [WLSMV Comparative Fit Index (CFI) = 0.96, Root Mean Square Error Of Approximation (RMSEA) = 0.05 (0.04–0.06), Standardized Root Mean Square Residual (SRMR) = 0.033], as such, we present the results for the MLR estimation for comparability with other models.

##### Model fit and selection

All indices used to assess model fit are summarized in Table 2. Guided by the literature (53, 54), thresholds of SRMR, CFI, and RMSEA were used as guidelines for quantifying global fit, rather than as strict rules (50, 55, 56). Only solutions that achieved acceptable or borderline global fit were summarized. Specific bifactor indices were calculated for the final model (i.e., asymmetrical bifactor) using the *BifactorIndicesCalculator* package (57) in *RStudio*.

**Table 2:** Indices and statistics used, along with thresholds and descriptors.

Index/Statistic	Purpose	Guideline	Descriptor	Reference
Factor-based methods				
Robust *χ*^2^	Absolute Fit	*p* > 0.05		(53, 54)
SRMR		<0.08		
Robust RMSEA	Approximate Fit	≤0.06		
Robust RMSEA 90% CI		0.10 not included in interval		
CFI		≥0.95		
Scaled χ^2^ difference tests	Model comparison	*p* < 0.05		(58)
AIC		Lower values indicate better fit		(59)
BIC				
Modification indices	Local fit	>3.84		
Standardized covariance residuals		< |1.96|		
Standardized factor loading	Convergent validity	>0.71	Excellent	(60)
		>0.63	Very Good	
		>0.55	Good	
		>0.45	Fair	
		>0.32	Poor	
Inter-factor correlations	Discriminant validity	<0.85		(59)
Omega coefficient (*ω* and *ω*_s_)	Total score and subscale-score reliability	>0.80	Good	(61)
		>0.70	Acceptable	(62)
Omega hierarchical coefficient (*ω*_H and_ *ω*_HS)_	Tenability of interpretation of a sole total score	>0.80		(63, 64)
ECV_ss_	Whether the use of subscales adds unique information			
ECV	Essential unidimensionality	PUC > 0.80, or ECV > 0.60 and *ω*_H > _0.70		(65)
PUC				
I-ECV	Common variance attributable to general factor in each indicator	>0.80–0.85		(66)
Composite-based methods				
Bootstrapped-based *d*_L_ and *d*_G_ (1,000 replications)	Global fit of the model (vs. saturated model)	<95% quantile of distribution		(21, 67)
Bootstrapped-based SRMR (1,000 replications)		<0.08		
RMS*_θ_*		<0.12		
Indicator weight magnitude	Local fit			
Indicator weight statistical significance		*p* < 0.05		
VIF	Multicollinearity and Suppressor Effects	<3.3		(68)
				(69)
Indicator correlations		>0.90	Very high	(70)
Composite correlations		0.70–0.90	High	
		0.50–0.70	Moderate	
		0.30–0.50	Low	
		0.00–0.30	Negligible	
*R*^2^ adj.	Explained variance	>0.25	Large	(71)
		>.09	Medium	
		>.01	Small	
*f* ^2^	Predictive validity	>0.35	Large	(71)
		>0.15	Medium	
		>0.02	Small	

CFI, Comparative Fit Index; RMSEA, Root Mean Square Error of Approximation; SRMR, Standardized Root Mean Square Residual; AIC, Akaike's Information Criteria; BIC, Bayesian Information Criteria; ECV, Explained Common Variance; PUC, Percentage of Uncontaminated Correlations; I-ECV, item ECV; RMS, Root Mean Square Error Covariance; *d*_L_, squared Euclidean distance; *d*_G_, geodesic distance; VIF, Variance Inflation Factor; CI, confidence interval.

#### Confirmatory composite analysis

Our initial postulated model conceptualized PL and its domains as composites. Therefore, we used *Confirmatory Composite Analysis* (CCA) (72) to mimic the analysis done through CFA and compare both measurement models.

**Figure 3:**
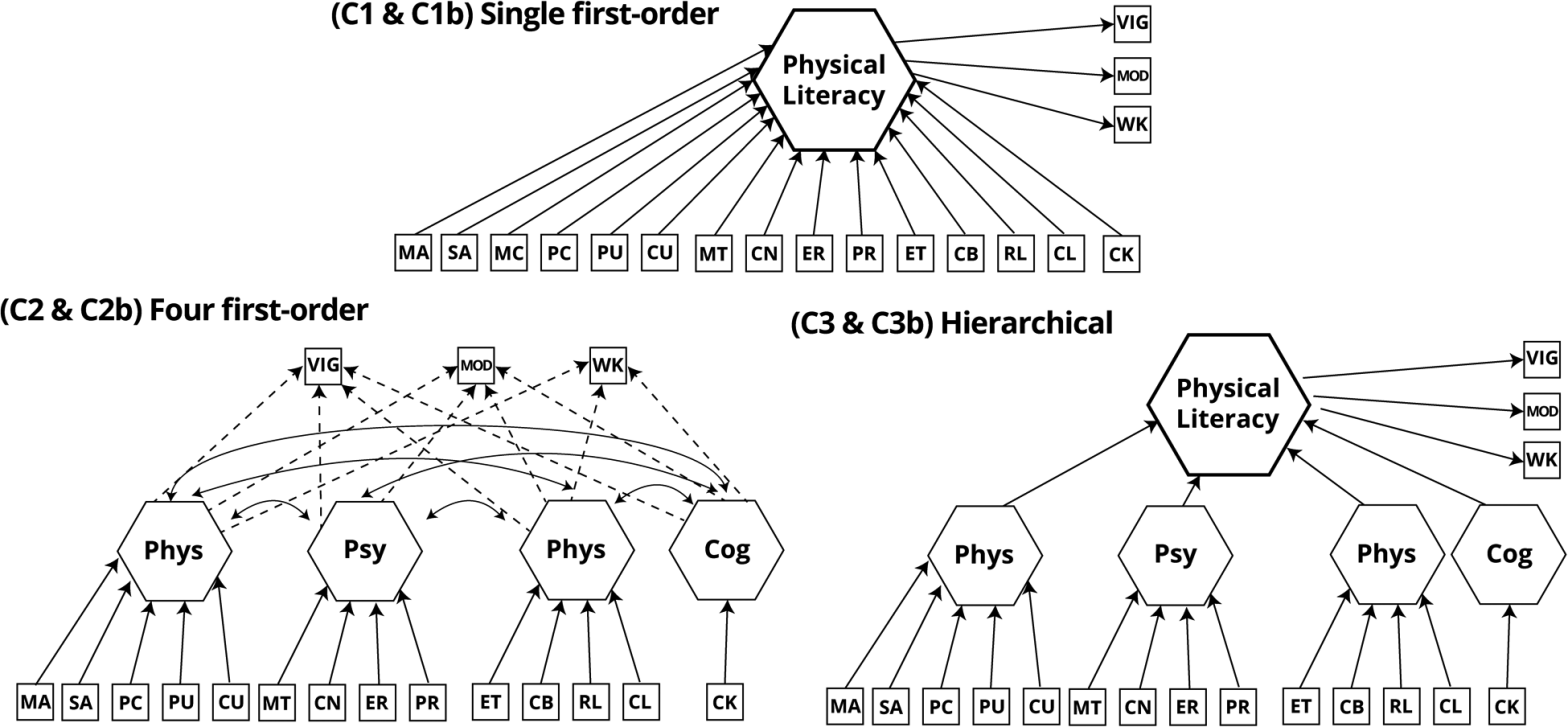
Composite-based models estimated in the study. PL, physical literacy; MA, manipulative-based activities; SA, stability-based activities; PC, PACER; PU, push-ups; CU, curl-ups; MT, motivation; CN, confidence; ER, emotional regulation; PR, physical regulation; ET, ethics; CB, collaboration; RL, relationships; CL, culture; CK, content knowledge.

All composite models were estimated in *cSEM* 0.4.0.9000 (73) using the PLS estimator with 1,000 bootstrap replications. All cases with missing data on any of the study variables were deleted (final *N* = 443) since no other options are available in *cSEM* at the time of writing. In parallel with the CFA analysis, three models were estimated in mode B (Figure 3). They included a single composite, correlated composite, and a second-order model of PL (using the “two-stage approach”) (74, 75). No bifactor model was estimated, since no literature exists to substantiate it in composite fashion.

To assess the impact of unit weights, models constrained to equal weights for each indicator in the respective composite were estimated. To identify the models, three single indicator factors (one for each intensity of self-reported PA) were inserted into the model as outcomes of the modeled composites. All indices used to assess model fit are listed in Table 2.

## Results

### Preliminary analysis

Bivariate correlations between all indicators displayed results compatible with the *a priori* factorial structure, i.e., indicators in the same domain correlated higher with indicators in the same domain than with those of other domains (Table 3). An exception to this was the correlations of the flexibility indicators (*Shoulder Flexibility* and *Sit-and-Reach*), which displayed either no correlation or low negative correlations with other indicators postulated to be in the Physical domain (*PACER*, *Push-ups*, *Curl-ups*, and Movement Competence factors). We removed these indicators from the following models.

**Table 3:** Pearson and polyserial bivariate correlation matrix.

Variable	1.	2.	3.	4.	5.	6.	7.	8.	9.	1.	11.	12.	13.	14.	15.	16.	17.	18.	19.	20.	21.
1. Self-reported PA–vigorous	—																				
2. Self-reported PA–moderate	0.38	—																			
3. Self-reported PA–walking	0.25	0.36	—																		
4. PACER	0.31	0.01	−0.04	—																	
5. Push-ups	0.29	0.03	−0.07	0.61	—																
6. Curl-ups	0.24	0.05	−0.03	0.44	0.41	—															
7. Shoulder stretch, right	−0.13	−0.02	−0.08	−0.03	0.08	0.24	—														
8. Shoulder stretch, left	−0.10	−0.05	−0.06	−0.02	−0.04	0.00	0.75	—													
9. Sit-and-reach, right	−0.09	0.05	−0.03	−0.14	−0.07	−0.05	0.31	0.32	—												
10. Sit-and-reach, left	−0.07	0.08	0.01	−0.14	−0.05	−0.01	0.32	0.30	0.93	—											
11. Manipulative-based activities	0.23	0.05	0.01	0.37	0.43	0.34	−0.23	−0.25	−0.23	−0.22	—										
12. Stability-based activities	0.18	0.06	−0.01	0.31	0.35	0.27	−0.06	−0.11	−0.03	−0.01	0.79	—									
13. Content knowledge	−0.01	−0.08	−0.07	0.17	0.10	0.10	0.19	−0.03	0.15	0.14	−0.06	−0.05	—								
14. Motivation	0.45	0.13	0.03	0.40	0.35	0.30	−0.05	−0.04	0.05	0.09	0.33	0.31	0.09	—							
15. Confidence	0.40	0.10	0.04	0.49	0.45	0.37	−0.08	0.02	−0.01	0.03	0.37	0.33	0.02	0.73	—						
16. Emotional regulation	0.06	0.06	0.05	0.19	0.21	0.11	−0.13	−0.04	−0.03	−0.02	0.16	0.14	0.03	0.28	0.42	—					
17. Physical regulation	0.24	0.10	0.14	0.28	0.19	0.25	−0.13	−0.09	0.05	0.06	0.20	0.17	0.006	0.50	0.56	0.44	—				
18. Culture	0.31	0.16	0.06	0.26	0.22	0.23	−0.10	0.07	0.10	0.10	0.21	0.20	0.10	0.47	0.46	0.16	0.36	—			
19. Ethics	0.03	0.03	0.13	−0.08	0.01	0.04	−0.03	0.03	0.11	0.13	0.05	0.04	0.11	0.21	0.16	0.23	0.32	0.16	—		
20. Collaboration	0.08	0.07	0.06	−0.13	0.01	0.02	−0.15	−0.09	0.05	0.07	0.04	0.01	0.04	0.23	0.19	0.17	0.32	0.23	0.63	—	
21. Relationships	0.21	0.10	0.13	0.07	0.07	0.07	−0.17	−0.05	−0.03	−0.01	0.13	0.14	0.03	0.36	0.39	0.20	0.39	0.34	0.42	0.63	—

PA, physical activity; PACER, progressive aerobic cardiovascular endurance run.

All correlations with Shoulder Stretch variables are polyserial correlations.

### Confirmatory factor analysis

#### Model fit

The bifactor S·I-1 model (F6) showed the best absolute fit (SRMR) and relative fit (CFI and RMSEA) to the data attaining an acceptable fit (see Table 4). The symmetrical bifactor model also achieved acceptable values in all indices. Despite not achieving acceptable fit by conservative standards, models F3 and F4 had fit indices close to more lenient standards of 0.90 for CFI, and borderline to RMSEA and SRMR of 0.08 (50).

**Table 4:** Model fit, mean factor loadings, and inter-factor correlations for factor-based models.

	First-order models			Second-order models		
Fit measure	Unidimensional (F1)	Correlated factors (F2)	Correlated factors (F3)^a^	Hierarchical (F4)	Bifactor (F5)	Bifactor S·I-1 (F6)
MLR *χ*^2^	1,217.07 (77), *p* < .001	675.20 (72), *p* < .001	329.15 (70), *p* < .001	378.38 (72), *p* < .001	182.02 (62), *p* < .001	160.62 (69), *p *< .001
Robust CFI	0.55	0.78	0.90	0.89	0.95	0.97
Robust RMSEA (90% CI)	0.18 (0.17–0.19)	0.13 (0.12–0.14)	0.09 (0.08–0.10)	0.09 (0.08–0.10)	0.07 (0.06–0.08)	0.05 (0.04–0.06)
SRMR	0.12	0.11	0.09	0.09	0.06	0.04
AIC	58,075.108	57,452.753	57,110.133	57,150.092	56,991.07	
BIC	58,253.850	57,652.773	57,318.665	57,350.112	57,233.65	
*χ*^2^ robust different test		F1 vs. F2: *χ*^2^ = 319.51, *Δ*df = 5, *p* = <.001	F2 vs. F3: *χ*^2^ = 7,338.7, *Δ*df = 2, *p *= <.001	F4 vs. F3: *χ*^2^ = 151.35, *Δ*df = 2, *p* = <.001	F3 vs. F5: *χ*^2^ = 712.34, *Δ*df = 8, *p* = <.001	
Mean factor loadings						
General					0.44 (0.20)	0.46 (0.20)
Physical			0.61 (0.15)		0.44 (0.16)	0.51 (0.17)
Psychological			0.69 (0.21)		0.32 (0.14)	0.30 (0.22)
Social			0.65 (0.22)		0.55 (0.36)	0.48 (0.37)
Cognitive			0.77		0.77	0.77
Factor correlations (SE)						
Physical–Psychological			0.67 (0.03)			0.48 (0.11)
Physical–Social			0.02 (0.06)			−0.33 (0.07)
Physical–Cognitive			0.19 (0.06)			0.14 (0.08)
Psychological–Social			0.42 (0.07)			−0.20 (0.12)
Psychological–Cognitive			0.06 (0.06)			−0.14 (0.09)
Social–Cognitive			0.09 (0.07)			0.00 (.06)

CFI, comparative fit index; RMSEA, root mean square error of approximation; MLR, maximum likelihood robust; SRMR, standardized root mean square residual; AIC, Akaike's information criteria; BIC, Bayesian information criteria; SE, standard error.

^a^ Correlated residuals between emotional regulation and physical regulation, and manipulative-based activities and stability-based activities.

There was an improvement in model fit for the baseline correlated factors (F2) over the unidimensional F1. Analysis of modification indices (MI) for the F2 model revealed several large values (largest MI = 372.25); however, only two theoretically plausible modifications emerged. The first was to free a residual covariance between both indicators of *Movement Competence* (*Manipulative-based activities* and *Stability-based activities*) as this might be due to a teacher's observation method factor. The second was between the *Emotional Regulation* and *Physical Regulation* indicators, which shared similarities in the wording of the items. We specified a *post-hoc* error covariance between these indicators, resulting in the F3 model. This model was kept for testing in further models. F3 was an improvement over F2 according to all indices.

MI analysis of F3 suggested the model could be further improved by allowing a cross-loading of the *Culture* indicator on the Psychological domain. While this might be theoretically defensible as some items in this scale deal with similar self-related concepts to those of the latter factor, we kept this parameter constrained since the following bifactorial specification would assess whether a general trait could best account for this implied correlation. Fitting the symmetrical bifactor solution (F5) resulted in an improvement over F3; however, MI analysis revealed that the largest MI (55.51) regarded a correlation between two group factors (Physical and Psychological, the two highest correlating first-order factors in the F3 solution). Therefore, the asymmetrical model was fit (F6), which resulted in better overall indices, lower MI, and most residuals below the 1.96 threshold. No direct comparison was possible due to the insertion of a global indicator of PL to estimate F6.

#### Convergent and discriminant validity

Model F3 mean factor loadings were very good, with correlations between factors ranging from 0.01 to 0.66 supporting discriminant validity. The correlation pattern was uneven, though, with the Psychological factor showing moderate correlations with only the Physical and Social factors.

Mean factor loadings in the group factors of the F5 and F6 solutions were lower than in F3. This was expected since, in these models, the group factors represent residual variance not explained by the general factor. Between these models, there was a marginal increase (*Δ* = 0.02) in mean factor loadings in the general factor, and a concomitant reduction in two of the group factors (i.e., Psychological and Social).

In the asymmetrical bifactor model (F6), three indicators had excellent loadings on the general factor (Physical Literacy), one had very good, one had good, and the remaining seven indicators had borderline (∼0.32) to poor loadings. *Content Knowledge* had no statistically significant loading (see Table 5). Loadings on the group factors (residual variance not explained by the general factor) ranged from 0.32 to 0.70, 0.04 to 0.58, 0.01 to 0.91, and 0.77, on the Physical, Psychological, Social, and Cognitive domains, respectively. Except for the indicators in the Psychological domain, all indicators had average higher loadings on their group factors than on the general factor (see Table 4). After accounting for the general factor (PL), half of the inter-factor correlations became negative, with the remaining three showing a decrease in their positive correlations.

**Table 5:** Item parameters and model-based indices for the bifactor S*1-1 model (F6).

Subtest	General		Physical		Psychological		Social		Cognitive		I-ECV
	Standardized loading (SE)	*S* ^2^	Standardized loading (SE)	*S* ^2^	Standardized loading (SE)	*S* ^2^	Standardized loading (SE)	*S* ^2^	Standardized loading (SE)	*S* ^2^	
PL global	0.76 (0.03)	0.58									1.00
Manipulative-based activities	0.30 (0.05)	0.09	0.41 (0.06)	0.17							0.35
Stability-based activities	0.30 (0.05)	0.09	0.32 (0.06)	0.10							0.46
PACER	0.41 (0.05)	0.16	0.70 (0.04)	0.49							0.25
Push-ups	0.35 (0.05)	0.12	0.67 (0.05)	0.45							0.21
Curl-ups	0.35 (0.05)	0.12	0.44 (0.05)	0.19							0.38
Motivation	0.74 (0.04)	0.55			0.31 (0.11)	0.10					0.85
Confidence	0.76 (0.04)	0.58			0.58 (0.08)	0.34					0.63
Emotional regulation	0.33 (0.06)	0.11			0.28 (0.08)	0.08					0.58
Physical regulation	0.68 (0.04)	0.47			0.04 (0.09)	0.00					0.996
Ethics	0.34 (0.06)	0.12					0.54 (0.04)	0.30			0.28
Collaboration	0.38 (0.06)	0.14					0.91 (0.06)	0.83			0.15
Relationships	0.54 (0.04)	0.29					0.47 (0.04)	0.22			0.57
Culture	0.57 (0.04)	0.33					0.01 (0.05)	0.00			0.999
Content knowledge	0.11 (0.05)	0.01							0.77 (0.02)	0.59	0.02
ECV/ECV_SS_		0.49		0.71		0.23		0.61		0.98	
*ω*/*ω*_S_		0.89		0.76		0.82		0.80		0.60	
*ω*_H_/*ω*_HS_		0.71		0.52		0.15		0.42		0.59	
PUC		0.79									

PL, physical literacy; *S*^2^, squared standardized loading; SE, standard error; PACER, progressive aerobic cardiovascular endurance run; ECV, Explained Common Variance; PUC, Percentage of Uncontaminated Correlations; I-ECV, item ECV; *ω*_S_, Omega coefficient subscale; *ω*_H/_*ω*_HS_, Omega hierarchical coefficient/Omega hierarchical subscale.

#### Variance and reliability

Regarding model-based reliabilities in the final model (F6), the total PL score (i.e., summing all indicators, after normalization) attained good reliability, being estimated that 89% of its variance was due to both the general and group factors (*ω* = 0.89). An estimated 71% of total score variance was due to individual differences in the general factor. Reliabilities for the subscale scores were all acceptable or good, except for the Cognitive score (*ω*_s_ from 0.60 to 0.82). Based on the relationship between *ω*_s_ and *ω*_Hs,_ three subscales attained the recommended thresholds for adding statistical value over and beyond that of the total score (recommended *ω*_Hs _= 0.212, 0.192, 0.192, and 0.244, respectively).

Similar results were estimated by the Explained Common Variance (ECV): 49% of the total common variance (inherent to both general and group factors) is explained by the general factor (see Table 5). Of all indicators, only three (*Motivation*, *Physical Regulation*, and *Culture*), achieved the tentative 0.80–0.85 threshold for item ECV (I-ECV) (66) and can be regarded as essentially being influenced by the general trait alone. Except in the Psychological domain, the most reliable variance in indicators was explained by their respective group factor, resulting in marked dimensional uniqueness (ECV_SS_ = 0.56, 0.22, 0.71, and 0.99; Physical, Psychological, Social, and Cognitive group factors, respectively). Based on ECV_ss_ and *ω*_S_, all group factors attain the recommended value for dimensional uniqueness (i.e., warrant interpretation) [recommended *ω*_S_ = 0.479, 0.815, 0.479, and 0.479; (63)]. Finally, 79% of all correlations were saturated by the general factor (Percentage of Uncontaminated Correlations, PUC = 0.79), bordering on the 80% recommendation (65) for consideration of essential unidimensionality in future SEM measurement models.

### Confirmatory composite analysis

#### Model fit

The single composite models (C1 and C1b) showed the best absolute fit to the data, with all estimated values below or bordering their critical value suggesting excellent fit to the data (see Table 6). Both C2 and C3 models provide an acceptable fit to the data, with estimated borderline SRMR (both cases) and RMS*_θ_* below their thresholds, despite having estimated global fit indices bordering above the critical value. In terms of unit-weighted models, summing every indicator with equal weights to produce a total score (C1b) reproduced the observed relationships in the model better than the alternative sum (also with equal weights per indicator) into domain scores (assuming each domain as an emergent variable; C2b).

**Table 6:** Model fit and inter-factor correlations for composite-based models (C1–C3b); *n* = 443.

Fit measure	First-order				Second-order	
	Single first-order composite (C1)	Single first-order composite–unit-weighted (C1b)	Four first-order composites (C2)	Four first-order composites–unit-weighted (C2b)	Hierarchical (C3)	Hierarchical–unit-weighted (C3b)^a^
RMS_θ_	<0.001	<0.001	0.04	0.03		
SRMR (critical value 95%)	0.050 (0.054)	0.059 (0.059)	0.078 (0.068)	0.087 (0.061)	0.071 (0.060)	0.090 (0.062)
*d*_L_ (critical value 95%)	0.391 (0.443)	0.534 (0.533)	0.928 (0.707)	1.147 (0.563)	0.767 (0.547)	1.228 (0.586)
*d*_G_ (critical value 95%)	0.085 (0.106)	0.118 (0.113)	0.179 (0.146)	0.215 (0.138)	0.169 (0.134)	0.224 (0.140)
Construct correlations						
Physical–Psychological			**0.49 (0.04)**	**0.47 (0.03)**		
Physical–Social			**0.27 (0.06)**	**0.12 (0.04)**		
Physical–Cognitive			**0.11 (0.05)**	0.06 (0.04)		
Psychological–Social			**0.53 (0.05)**	**0.49 (0.04)**		
Psychological–Cognitive			0.07 (0.05)	0.05 (0.05)		
Social–Cognitive			0.05 (0.05)	0.09 (0.05)		

RMS, root mean square error covariance; SRMR, standardized root mean square residual; *d*_L_, squared Euclidean distance; *d*_G_, geodesic distance.

Statistically significant weights (*p *< 0.05) are bolded.

^a^ Only the first-order composite is produced by summing the indicators with equal weights; total score for hierarchical score is obtained by optimally weighting its first-order scores.

#### Variance

Standardized weights in the single composite model (C1; Table 7) ranged from −0.25 to 0.61, with *Motivation* being the only indicator with a statistically significant result. All other indicators do not contribute beyond this indicator. High correlations (<0.70) existed between some indicators, with corresponding Variance Inflation Factor (VIF) ranging from 2.26 to 3.14, suggesting the existence of suppressor effects.

**Table 7:** Item parameters and total effects of single first-order composite models (C1 and C1b); *n* = 443.

Composite ← indicators	*β* (SE)	VIF	Indicator correlation
PL←			−0.14 to 0.77
Manipulative-based activities	0.20 (0.14)	2.88	
Stability-based activities	−0.25 (0.13)	2.55	
PACER	0.15 (0.16)	2.03	
Push-ups	0.12 (0.15)	1.89	
Curl-ups	0.09 (0.10)	1.39	
Motivation	**0.61 (0.13)**	2.39	
Confidence	0.13 (0.10)	3.14	
Emotional regulation	−0.17 (0.10)	1.41	
Physical regulation	0.03 (0.13)	1.84	
Ethics	−0.07 (0.12)	1.76	
Collaboration	−0.05 (0.14)	2.36	
Relationships	0.15 (0.12)	1.89	
Culture	0.18 (0.11)	1.38	
Content knowledge	−0.14 (0.10)	1.10	
Optimal weights (C1)	Total effects β (SE)	*f* ^2^	*R*^2^ adj.
IPAQ ← PL			
Vigorous	**0**.**52** (0.03)	0.36	0.27
Moderate	**0.16** (0.06)	0.03	0.02
Walking	0.07 (0.07)	0.00	0.00
Unit weights (C1b)			
IPAQ ← PL			
Vigorous	**0.39 (0.04)**	0.18	0.15
Moderate	**0.12 (0.05)**	0.02	0.01
Walking	0.07 (0.05)	0.01	0.00

*β*, standardized weights; SE, standard error; VIF, variance inflation factor; *R*^2^ adj., adjusted *R*^2^; PACER, progressive aerobic cardiovascular endurance run; PL, physical literacy; IPAQ, international physical activity questionnaire.

Statistically significant (*p* < 0.05) weights are bolded.

In the correlated composites model (C2; Table 8), standardized weights increased for most variables. Six indicators had non-statistically significant weights and two were borderline (*p* ≈ 0.05). VIF values at indicator level were lower, with all composites showing high correlations among some of its indicators, with unexpected, inverted signs in three. Correlations among composites ranged from negligible (0.05) to moderate (0.53) (70) (see Table 6), with correlations with the Cognitive composite being all negligible and non-statistically significant (0.05–0.11).

**Table 8:** Item parameters and total effects of four first-order composite models (C2 and C2b); *n* = 443.

Composite ← indicators	*β* (SE)	VIF	Indicator correlation
Physical←		1.33	0.29–0.77
Manipulative-based activities	**0.41 (0.20), *p *= 0.050**	2.82	
Stability-based activities	−0.23 (0.19)	2.50	
PACER	**0.53 (0.17)**	1.72	
Push-ups	0.28 (0.19)	1.79	
Curl-ups	0.22 (0.15)	1.34	
Psychological←		1.70	0.29–0.73
Motivation	**0.70 (0.13)**	2.22	
Confidence	**0.40 (0.15)**	2.57	
Emotional regulation	**−0.22 (0.11), *p *= 0.065**	1.35	
Physical regulation	0.06 (0.15)	1.64	
Social←		1.40	0.13–0.63
Ethics	−0.02 (0.22)	1.64	
Collaboration	−0.24 (0.24)	2.22	
Relationships	**0.59 (0.17)**	1.69	
Culture	**0.76 (0.14)**	1.12	
Cognitive←		1.01	—
Content knowledge	1.0		
Optimal weights (C2)	Total effects β (SE)	*f* ^2^	*R*^2^ adj^a^.
IPAQ ← Physical			
Vigorous	**0.19 (0.05)**	0.04	0.25 0.04 0.02
Moderate	−0.04 (0.06)	0.00	
Walking	−0.08 (0.07)	0.01	
IPAQ ← Psychological			
Vigorous	**0.36 (0.05)**	0.10	
Moderate	0.08 (0.06)	0.00	
Walking	0.04 (0.07)	0.00	
IPAQ ← Social			
Vigorous	0.06 (0.05)	0.00	
Moderate	**0**.**15 (0.06)**	0.02	
Walking	0.13 (0.08)	0.01	
IPAQ ← Cognitive			
Vigorous	−0.04 (0.04)	0.00	
Moderate	−0.08 (0.05).	0.01	
Walking	**−0.08 (0.04)**	0.01	
Unit weights (C2b)			
IPAQ ← Physical			0.17 0.02 0.03
Vigorous	**0.22 (0.05)**	0.04	
Moderate	−0.00 (0.06)	0.00	
Walking	−0.09 (0.06)	0.00	
IPAQ ← Psychological			
Vigorous	**0.25 (0.05)**	0.05	
Moderate	0.11 (0.06)	0.01	
Walking	0.08 (0.07)	0.00	
IPAQ ← Social			
Vigorous	0.03 (0.06)	0.00	
Moderate	0.08 (0.07)	0.01	
Walking	0.12 (0.06)	0.01	
IPAQ ← Cognitive			
Vigorous	−0.02 (0.05)	0.00	
Moderate	−0.08 (0.04)	0.01	
Walking	−0.09 (0.05)	0.01	

*β*, standardized weights; SE, standard error; VIF, variance inflation factor; *R*^2^ adj., adjusted *R*^2^; PACER, progressive aerobic cardiovascular endurance run; PL, physical literacy; IPAQ, international physical activity questionnaire.

Statistically significant (*p* < 0.05) and borderline weights are bolded.

^a^Results displayed refer to the combined effect on vigorous, moderate, and walking intensities, respectively.

Weights, VIF, and indicator correlation mostly kept their magnitude in the second-order composite (see Table 9). The exception was an increase in weights for *Push-ups*, *Curl-ups*, and *Physical Regulation* indicators, and a decrease in *Emotional Regulation* and *Manipulative-based Activities*. First-order weights on the second-order composite attributed a higher relative contribution to the Psychological composite (*β* = 0.69), in explaining variance in the second-order composite of PL. Analysis of the first-order loadings (bivariate correlations) suggested that the Physical and Social composites (loadings = 0.68, not shown) were still important in composing this second-order composite, despite explaining substantially fewer amounts of variance.

**Table 9:** Item parameters and total effects of the hierarchical composite models (C3 and C3b); *n* = 443.

Composite** ← **indicators	*β* (SE)	VIF	Indicator correlation
First-order			
Physical ←			0.29–0.77
Manipulative-based activities	0.19 (0.14)	2.82	
Stability-based activities	0.09 (0.14)	2.50	
PACER	**0.54 (0.11)**	1.72	
Push-ups	**0.21 (0.12) *p* = 0.072**	1.79	
Curl-ups	**0.29 (0.10)**	1.34	
Psychological←			
Motivation	**0.44 (0.08)**	2.22	0.29–0.73
Confidence	**0.56 (0.10)**	2.57	
Emotional regulation	−0.10 (0.07)	1.35	
Physical regulation	**0.16 (0.08), *p* = 0.06**	1.64	
Social←			0.13–0.63
Ethics	0.08 (0.12)	1.64	
Collaboration	−0.14 (0.14)	2.22	
Relationships	**0.55 (0.09)**	1.69	
Culture	**0.73 (0.07)**	1.12	
Cognitive←			—
Content Knowledge	1.0	—	
Second-order			
PL←			
Physical	**0.27 (0.13)**	1.42	
Psychological	**0.69 (0.13)**	1.93	
Social	0.22 (0.15)	1.48	
Cognitive	−0.14 (0.10)	1.02	
Optimal weights (C3)	Total effects *β* (SE)	*f* ^2^	*R*^2^ adj.
IPAQ–PL			
Vigorous	**0.48 (0.04)**	0.30	0.23
Moderate	**0.16 (0.05)**	0.03	0.02
Walking	0.09 (0.06)	0.01	0.01
Unit weights (C3b)			
IPAQ–PL			
Vigorous	**0.41 (0.04)**	0.20	0.17
Moderate	**0.14 (0.05)**	0.02	0.02
Walking	0.09 (0.06)	0.01	0.01

*β*, standardized weights; SE, standard error; VIF, variance inflation factor; *R*^2^adj., adjusted *R*^2^; PACER, progressive aerobic cardiovascular endurance run; PL, physical literacy; IPAQ, international physical activity questionnaire.

Statistically significant (*p *< 0.05) weights and borderline weights are bolded.

All optimally weighted approaches (C1, C2, and C3) explained similar amounts of variance in self-reported vigorous PA, with the single composite model having marginally higher values (*R*^2^ adj. = 0.27; Table 7). The variance explained on moderate and walking self-reported PA was negligible. Using the correlated composite (C2) approach revealed different contributions by composite; Psychological and Physical domains had a higher effect size (*f*^2^) on vigorous PA, while the Social domain had a low effect size on moderate PA (71). Unit-weighting produced reductions in all effect sizes compared to optimally weighted composites, with the greatest reduction in the single composite (C1b). Again, the correlated composites model (C2b) revealed a sharper decrease in the contributions of the Psychological composite, with others maintaining their relative magnitudes.

## Discussion

This paper aimed to assess the construct validity of the PPLA and the adequacy of using a PL total score and respective subscores. It also investigated the practical and ontological implications of the different methods for PL. In summary, results from factor-based methods suggested that an asymmetrical bifactor model with correlated group factors (F6) provided the best fit to the data. This suggests the existence of a common trait underlying reliable individual variation of response, simultaneous with significant uniqueness in each domain. In composite-based methods, results suggested that the single first-order composite models (C1 and C1b) provided the best fit, while the hierarchical model (C3) provided the most interpretable solution for comparison purposes. Overall, evidence supported the construct validity and reliability of the PPLA for its intended use as an integrated tool to measure PL as a multidimensional construct in 15- to 18-year-old Portuguese students in a physical education setting, with comparable results across both factor-based and composite-based methods.

### Factor-based methods

Our results from factor-based methods suggest that the best-fitting representation of a measurement model for the PPLA is an asymmetrical bifactor model (F6) with correlated group factors. These findings differed from those found in other PL measuring batteries. In the most recent construct validation effort of the *Canadian PL Assessment* (CAPL), a second-order factor was modeled to account for correlations between domains of PL (10). However, the authors did not report fitting a bifactorial model. Similarly, in a validation of a PL measuring model for children and youth (24), a second-order factor model was chosen as the best representation of the data (with a bifactorial model providing an inadmissible solution). In our study, estimation of a second-order factor model provided a worse fitting (compared to both a correlated factors model and bifactor models) and an inadmissible solution to the data.

We gather that this might stem from an artifact produced by an uneven pattern of correlations among factors, which does not suggest a direct underlying common cause (i.e., factor). While the Physical factor correlated highly with the Psychological factor and moderately with the Cognitive factor, it did not correlate sufficiently with the Social factor. Despite using different measures and operational definitions of the constructs, the CAPL's correlations among factors followed a similar pattern, which then resulted in one of the posited first-order factors (*Knowledge and Understanding*) having a poor loading (0.21) on the second-order Physical Literacy factor (10). Similar results emerged in our study, providing evidence against a second-order model interpretation, with the first-order factor mediating the effect of PL on each indicator. A bifactorial model represents direct effects of the general factor (PL) on indicators, with the asymmetrical version allowing for correlations among group factors, resulting in a better fit than that of its symmetrical counterpart. This suggests that the orthogonality constraint was overly restrictive and that the PL general factor fails to account for all shared variance among domains.

Our results from the bifactor model (F6) analysis suggest the existence of a common trait underlying reliable individual variation of responses (i.e., Physical Literacy), albeit not with the strength required for a meaningful statistical interpretation of a total-PPLA score in isolation. Instead, the complementary use of unit-weighted subscale scores has added value over and beyond the single total score since they present enough dimensional uniqueness. A noteworthy exception is that of the interpretation of the Psychological subscale. This occurs since indicators in this domain seem to be saturated by the general factor. Any interpretation of differences on this subscale would be biased by shared variance across domains. A tentative interpretation of this fact can be given by the prominent role of psychological variables in predicting PA in both adolescents (76, 77) and adults (78); similarly, these variables might play a mediating role between other domains.

Similarly, despite achieving borderline values to be considered essentially measuring the single trait of PL, high values of ECV_SS_ and moderate values of ECV suggest that further research in SEM contexts should use a bifactor measurement model for the PPLA. This would also allow testing of different effects of the general factor and group factors.

### Comparison with composite-based methods

The single composite models (both optimally and unit-weighted) attained the best fit, with optimally weighted first-order and hierarchical factor (C2 and C3) providing borderline adequate approximate fit to the data. Of these, the latter provided the most interpretable solution in terms of individual contribution of indicators, since it reduces the possibility of multicollinearity. Despite attaining excellent fit by all metrics, the single optimal-weighted model (C1) had non-statistically significant weights for all but the *Motivation* indicator. This could be explained by the existence of high correlations among indicators and the number of indicators estimated in the same composite.

Although no assumptions regarding covariation of the indicators are made in a composite model, high correlation patterns among indicators will generally result in multicollinearity and cause suppression effects, co-occurrence of positive and negative weights (i.e., “flipped signs”), and preclude a meaningful interpretation of these weights in general (69). Since a multiple regression is used to estimate the weights of indicators, these are competing for explained variance, increasing the chance of non-statistically significant weights to be estimated. This phenomenon was minimized in the correlated composites, and mainly in the hierarchical model (C3), where most indicators had statistically significant (or borderline) weights, with the expected direction.

Comparing across methodologies, both the hierarchical composite model and asymmetrical bifactor model attained similar results. In the former, both *Manipulative-based* and *Stability-based Activities*, along with *Emotional Regulation*, *Ethics*, and *Collaboration* indicators, did not contribute to explaining variance in their respective composites over and beyond other indicators. While the magnitudes of standardized loadings in the latter obtained by these indicators in the general factor of PL, along with I-ECV, were poor. A similar case occurred with the *Content Knowledge* indicator. Its poor performance was carried into the first-order weight. Analyzing the first-order weights and the inverse of the ECV_SS_ suggests a similar pattern in that the Psychological indicators contribute more to the general factor/higher-order composite (*β* = 0.69, ECV_GS_ = 0.78). whereas the Physical (*β* = 0.27, ECV_GS_ = 0.29) and Social (*β* = 0.22, ECV_GS_ = 0.39) indicators contribute similarly, and the Cognitive indicator contributes marginally (*β* = −0.14, ECV_GS_ = 0.02). This is parallel to our earlier discussion on the absorption of the most of the Psychological indicators’ variance into the general PL factor (asymmetrical bifactor model).

Thus, these models could be further improved by dropping indicators with statistically nonsignificant weights (69), indicators with low I-ECV, or with poor loadings on both the general and group factors (64). This, however, might compromise content validity of the PPLA and meaningful interpretation of these indicators within their group factors. We recommend that before any removal of indicators is undertaken, this analysis should be replicated in a large independent sample and outside of COVID-19 restrictions, which might change the effects on how different elements of PL correlate with each other and concomitantly on the measurement models. Future development of instruments to measure the remaining elements of the Cognitive domain (i.e., *Tactics* and *Rules*) might draw a different global picture for the construct.

Further parallels can be drawn between results in the different methodologies. In both correlated factors models, correlations among the different domains maintained a similar relative pattern. The Psychological domain was moderately correlated with the Physical and Social domains, with the remaining correlations being lower. A noteworthy difference is an increase in correlation among the Physical and Social domains in the composite model, which could be attributed to a difference in indicator weighting between models.

In conclusion, evidence in favor of a measurement model with a higher-order PL construct (either represented by an asymmetrical bifactor or composite hierarchical model) was mostly robust across methods, with comparable results. Regarding the use of a total summed score, the methods present slightly different results. In the composite-based methods, total score was an adequate representation of an emergent PL variable, while in the factor-based methods, this total score does not quite reach the uniqueness (*ω*_H_) needed to represent a singular latent variable. Based on this, we advise calculation of a total summed PL score, along with domain scores, which should be interpreted conjointly in applied settings.

### Conceptual implications for physical literacy

From a reflective perspective, a bifactor view seems the most empirically and conceptually plausible one, since it is tenable that transversal broadband meta-learning or disposition influences all different elements in a movement context, while domain-specific processes inherent to the different physical, affective, social, and cognitive skills originate clusters of highly interdependent variance. This seems compatible with the APLF's conceptualization of a higher learning state where learning in one element is transferable between elements and domains [*Transfer and Empowerment*, akin to the *Relational Abstract* level of the Structure of Observed Learning Outcomes (79)].

Also, if a higher-order common factor perspective is tenable, then different domains and elements are theoretically interchangeable since they are merely a sample of the infinite indicators and facets that could be chosen to measure PL. While it seems plausible that a different set of indicators could be selected according to the research questions and applications at hand, it might diminish the integrated perspective that researchers have been seeking all along if proper care is not taken to ensure representation across all domains. The asymmetrical bifactor model, however, offers a compromise solution as it becomes possible to acknowledge that while indicators are interchangeable within a domain, domains themselves are not interchangeable (80, 81) and are essential to defining the PL construct. Other plausible interpretations include that of PL as a network of interconnected latent variables that may or may not correlate [i.e., similar to our F3 model, and initial efforts of the CAPL (23)]. This could, however, compromise the place of PL in educational policy discussion, since it would present no added value as a whole variable and could easily be dissected based on convenience.

From a diametrical perspective, viewing PL as an emergent variable through a pragmatic lens (i.e., as a composite, assumed without measurement error or disturbance terms) could also be plausible. As such, rather than being an existing phenomenon to be measured, PL would instead be an umbrella term to designate and index a nomological network of variables that form a sum higher than its individual parts to predict movement-related outcomes throughout the life course, without a singular common cause (82, 83). This would also recognize that selected indicator variables might share a distal common cause mediated through a complex chain of mediators and moderators. This is a vision more compatible with the epistemic phenomenology position of the *Whiteheadian* school of thought, wherein everyone might have a different pattern of correlations (including no correlation) among domains and elements depending on their personal understanding and development of PL. A risk, however, to this interpretation is the possibility of interpretational confounding with data-derived weights (optimal weights), which could compromise the theoretical standing of the concept and similarly hinder meaningful progress in educational practices if care is not taken in interpretation and dissemination. A solution for this might be the use of unit-weighted composites (as shown), or *a priori-defined* weights based on theory or intended usage.

Alternatively, a causal-formative framework could also be used, given PL's composite-based nature (22, 67). The scope of PL, in this case, would be directly defined by its composing domains and would require that all domains of PL be included when estimating the model, which would reinforce its holistic nature. This would view PL as an aggregated latent variable composed of multiple non-exchangeable domains whose variation could be explained by variation in only a specific set of elements that did not mandate concomitant variation in all elements (22, 83). Further research should seek to reconcile and/or discuss these paradigms.

Since our results are compatible with both the common factor and composite interpretations, we take a practical stance and tentatively recommend the common factor lens of analysis. This is the implicit foundation of both Classical Test Theory (CTT) and IRT which afford access to a robust analysis toolkit (e.g., FIML estimation) to explore dimensionality, score adequacy, and response patterns (17–19). It may also afford the possibility of disentangling the impact of different group factors on intended outcomes of PL by controlling for the general PL factor. Nonetheless, further comparison of practical impacts on derived scores, with different datasets and under different conditions, might be warranted to determine the adequacy of the conceptual interpretation described.

### Strengths and limitations

A major strength of this study was the comparison between two different methodologies to draw inferences about the construct validity and reliability of the PPLA. Second, to our knowledge, this is the first study to demonstrate the application of bifactor models to a PL assessment tool that assessed the adequacy of interpretation or use of scores and subscores. Third, our study builds upon measures that have gathered evidence of construct validity and reliability at item-level using Item Response Theory methodologies that provide accurate estimates, and the capability to study item quality and psychometric behavior.

Some limitations of our study include *post-hoc* modifications to initially hypothesized models (i.e., correlated residuals) and the need to use bounded estimation for the factor-based higher-order models. We did not account for multilevel grouping within data (i.e., schools and classes), which could also hold some bias over the results.

Despite mimicking the relative composition of grade 10–12 student population in Portugal according to both grade and course major, our sample was a convenience one. All these points warrant caution before generalizing any of our findings outside of this sample, without further cross-validation with a larger independent sample and multilevel estimation. This is also a requirement if scores derived from PPLA are used as antecedent or precedent variable(s) in extended studies.

Similarly, to assess whether studied relationships among constructs hold across different population groups, measurement invariance should be assessed for the full model, along with its predictive validity on meaningful outcomes (e.g., objectively measured PA, wellbeing), which was not a focus in this study. We also recognize that PL could cogently be modeled using equivalent or alternative models and encourage further research.

Another limitation created using IRT-calibrated measures at indicator level was the incapability to account for measurement error at the lower abstraction level (which is one of SEM's strengths). This could have attenuated correlations among first-order factors and biased our overall interpretations. Future methods to account for this should be used.

A particular conceptual limitation was the elimination of flexibility indicators in this version of the PPLA. We argue these indicators are relevant to the whole-picture PL and their inclusion should be considered and scrutinized in future efforts.

## Conclusion

Using both confirmatory factor analysis and confirmatory composite analysis, we gathered evidence supporting the construct validity and reliability of the PPLA as an integrated tool to measure PL as a multidimensional construct in 15- to 18-year-old Portuguese students. Out of all the estimated models, the bifactor model enabled richer conclusions on the tenability and interpretation of total and subscales (per PL domain) scores. Nonetheless, a composite model description also seems preliminary tenable and useful for predicting self-reported PA. Present results provide evidence that a general trait of PL accounts for a considerable amount of variance in all indicators, albeit with insufficient strength to be interpreted in isolation, along with clear domain-specific variance.

Based on this, we suggest calculation of a total summed PL score, along with domain scores, which should be interpreted conjointly in applied settings. While the former provides a heuristic summary for a quick comparison of different classes and schools in low-stakes settings, the latter allows for a more meaningful interpretation of students’ PL profiles and needs. We also recommend the use of total scores per element/indicator in contexts that would benefit from the detail they provide. Most research settings would benefit from using a bifactor measurement model, which enables disentanglement of the variance attributed to the general PL trait and its domains.

We believe all these options will offer flexible solutions for both practitioners—generating specific feedback for students, families, teachers, and schools—and researchers—supporting, e.g., efforts to monitor quality PE practices and the longitudinal impact of educational policies and/or specific interventions on PL. This will contribute to a better understanding of the development of PL and, ultimately, more meaningful PL journeys.

## Data Availability

The datasets presented in this article are not readily available because participants of this study did not explicitly agree for their data to be shared publicly. Requests to access the datasets should be directed to the corresponding author.
