# Supplementary material for: Portuguese Physical Literacy Assessment for adolescents (15–18 years): validation using confirmatory factor and composite analyses
Source: Front Sports Act Living. 2023 Jun 27;5:1192025. doi: 10.3389/fspor.2023.1192025 (PMC10333539; doi:10.3389/fspor.2023.1192025)
Supplement: Supplementary file 1 [file Table1.docx]

# Supplemental Material 1 - Literature Review

## Measuring model conceptualization

While most construct validation studies and scale development done in education and social sciences have been under the reflective paradigm (1), many disciplines are now starting to explore the formative paradigm (2,3). This has generated an ongoing debate and research on the latter’s impact and adequacy for measurement (4–9). Each of these conceptualizations bears different ontological and practical premises.

### Reflective measurement

#### Ontological and conceptual issues

Measurement under the reflective conceptualization assumes a realist perspective (10), wherein the construct represents a real entity tangible through its measures. It is represented through a common factor underlying all observed measures (effect indicators) and is responsible for their covariance (11). This is the approach at the heart of Classical Test Theory (CTT) (also named True Score Theory) (12) and Item Response Theory (13,14). According to this, a causal relationship is assumed between the construct and its measures, with a change in the construct expected to cause equivalent changes (after accounting for error) in all its measures (2). The notion of measurement error is explicitly modeled at indicator-level (i.e., residuals) which is then used in factor-based Structural Equation Modelling (SEM) methodologies to provide more accurate depictions of correlation among constructs (i.e., through diattenuation; R. B. Kline, 2016)

Factors (i.e., latent variables) are assumed unidimensional entities, composed of multiple interchangeable measures of the same phenomenon; and elimination of any of these does not alter the meaning of the factor (2). Testing of this dimensionality structure is usually performed using covariance-based methods like Confirmatory Factor Analysis (CFA), by comparing different *a priori* specifications based on substantive theory (2). Constructs, however, are unlikely to adhere to strict unidimensionality, whether intentionally - because the researchers wanted to capture different facets of the construct through to the use of subscales – or unintentionally – due to method factors (2), responses bias (16) or even item difficulty/popularity (17). As such, a multidimensional model can be estimated, with the most common one being the a) correlated first-order factor models, and b) higher-order models (18,19). Other options include the estimation of correlated residuals between indicators to accommodate covariation unrelated to the construct at hand (2).

Correlated factor models freely estimate correlations among constructs composed of interchangeable indicators (c.f., Figure 2 in the main file). If the pattern and magnitude of correlations are substantial, and there is a strong conceptual justification, it is then tenable to estimate a higher-order model to account for this correlation (15,20). The two most common options for this are the hierarchical model and the bifactor model.

In both cases, the different first-order factors are conceptualized as being interchangeable indicators of the same, more abstract, construct (21). One of the main differences lies in the way the effect of the higher-order factor is conceived upon observed indicators: in the hierarchical model, the first-order factor fully mediates the effect of the higher-order construct on the indicators (c.f. model F4 in Figure 2, in the main file) while in the bifactor model, direct effects are estimated from the higher-order construct into the indicators (22,23). Bifactor model allows the analysis of group-factors (equivalent to the error terms on the hierarchical model, denominated disturbances) (18) which represent variance that is specific to a set of indicators, over and beyond that of the general factor (i.e., the higher-order construct); and modeling of independent relationships of the group and general factors on outcomes of interest (22,24). Because of these nuances, the bifactor model enables a more robust study of dimensionality and the interpretation of scores, which we will describe below.

Further iterations of the bifactor model have been developed as this approach gains traction within measurement assessment of many disciplines; namely non-symmetrical bifactor models - bifactor S-1 or S·I-1 models (25,26). These can be used when the group factors are not interchangeable indicators (i.e., *random effects*), and are structurally different (i.e., *fixed effects*) – as is usually the case when researchers conceptualize facets or domains. These allow for estimation of correlation among group factors – which in the canonical bifactor model are constrained to zero by definition (27) – and use a group factor or general measures of a construct to establish a reference frame based on previous theory, instead of allowing for data’s intricacies to do so by collapsing specific indicators or entire group factors – anomalies which are usual in the estimation of canonical bifactor models (26).

#### Scoring implications

Summation of indicators to obtain an approximation of the position of each individual on the latent variable is one of the most common uses for scales and measurement instruments in applied settings (28–30). This assumes that all indicators are equally weighted (i.e., unit-weighted), and represents one of many unrefined approaches to derive weights (31). Other options include optimal weighting (i.e., using loadings, or factor coefficients derived from CFA), and refined approaches which use all available information to produce factor scores. However, due to specific nuances of the common factor model (i.e., factor indeterminacy) (32), extracted factor scores might not be adequate for individual-level analysis or interpretation. Despite being a simple, viable, and robust alternative in many settings (12,33), sum-scores’ adequacy rests on the dimensionality of the scale or instrument in question (34).

When there is evidence of a strong factor underlying the results (with high indicator loadings), then the correlation of the derived total sum-score with the *true* factor score will be high. Otherwise, the use of scores for each of identified domains (as in the correlated factors model) might be justified (35). To assess the tenability of a total score, along with eventual subscales scores, a bifactor model can be fit, and various model-based indices derived (34,36,37). These allow the researcher to evaluate the amount of variance accounted for by the general factor and group-factors (specific domain), and whether their strength warrants statistical or empirical interpretation. It also allows testing whether the use of a unidimensional model in SEM settings would adequately convey the general trait, or whether a bifactor model should be fit.

### Formative measurement

#### Ontological and conceptual issues

Two different views exist within this perspective according to the conceived ontology of the construct, causality, error, conceptual unity of its indicators, and subsequent estimation: a) causal indicators, and b) composite indicators (13). We will first address their differences and later describe their similitudes.

##### Causal indicators

Similar to the reflective approach, a latent variable is still posited to *exist*, however, instead of it accounting for the variation in its indicators, causal indicators form, or influence the latent variable (2,3). Included indicators of a construct must share conceptual unity (i.e., pertain to the same concept), and must cover all possible content domains of the construct (38). Measurement error is conceived at the construct-level through the estimation of a disturbance term, and it is posited to account for possible unincluded indicators or facets (13,39).

Estimation of this type of model can only be achieved in covariance-based methods (i.e. CFA or factor-based SEM) (40) by specifying two emitted paths from each causal-formative variable (41) – either intended outcomes or direct reflective measures of the same construct (creating a multiple indicators-multiple causes model).

##### Composite indicators

Conversely to both earlier approaches, constructs defined by composite indicators (i.e., emergent variables, composites, or artifacts; Henseler, 2021) have no ascribed existence independent of measurement (constructivism) and thus, are created for mostly analytic purposes (operationalism) (4,10). Included indicators need not share conceptual unity (13), and are assumed to completely define the construct (42) since no error term is estimated– neither at item, nor construct-level (c.f., Figure 3 in the main file).

Composite estimation is best done through variance-based methods (i.e., composite-based) (40), and one of its many available estimators – with the most studied estimator being Partial Least Squares (PLS) (43). In this framework, identification of the composite requires only a connected construct (i.e., non-isolation condition) (43).

##### Similitudes

Despite the aforementioned differences, both formative approaches share the fact that constructs are estimated as addictive - i.e., a linear combination of weighted indicators (20). These constructs are multidimensional by definition, formed by indicators that each capture a non-redundant facet of the concept; as such, removal of any indicator will change the meaning of the estimated construct (13,39,42). These constructs are not limited to a single first-order conceptualization and can take a similar multitude of structures implied by the reflective measurement paradigm, including a) correlated first-order and b) higher-order models (44).

Theoretically, no degree of correlation among indicators is required (38,42), since a change in one indicator might not be accompanied by change in all indicators (28); in practice, high levels of correlations among indicators can cause issues of multicollinearity that difficult interpretation of parameters (45,46).

#### Scoring implications

The main difference regarding scoring in formative models is that the score loses its conceptualization as a position on a posited trait, and is rather equated to an index – a summary of data reduction (10). As such, a simple sum-score might provide a parsimonious estimate, at the cost of distinct information in each indicator (47), especially when correlations among indicators are low (48). As in the reflective model, usage of differential weights might be an option to address this issue. An advantage of composite-based methods is the inherent determinacy of construct scores (49) – since they represent linear combinations of weighted estimates. As such, weighting indicator scores by their regression weights will be equivalent to the estimated construct scores.

Another implication, albeit disputed (50), is that of susceptibility to interpretational confounding in weight estimates – i.e., difference in the weights attributed to each indicator depending on the variables used to identify the model (48,51–53). This is also argued to compromise theoretical development (54) and meaningful interpretation of the construct (55) since the same construct might change depending on the nomological network into which it is inserted. To resolve this issue, some researchers suggest the use of predetermined weights based on theory (21,28) or revert to the simpler solution: unit weights (42,46,56).

## References

1. Bollen KA. Latent Variables in Psychology and the Social Sciences. Annu Rev Psychol. 2002 Feb;53(1):605–34.

2. Brown TA. Confirmatory factor analysis for applied research. Second edition. New York ; London: The Guilford Press; 2015. 462 p. (Methodology in the social sciences).

3. Diamantopoulos A. Formative indicators: Introduction to the special issue. J Bus Res. 2008 Dec 1;61(12):1201–2.

4. Edwards JR. The Fallacy of Formative Measurement. Organ Res Methods. 2011 Apr;14(2):370–88.

5. Evermann J, Rönkkö M. Recent Developments in PLS. Commun Assoc Inf Syst. 2021;44:123–33.

6. Henseler J. Partial least squares path modeling: Quo vadis? Qual Quant. 2018 Jan 1;52(1):1–8.

7. Henseler J, Dijkstra TK, Sarstedt M, Ringle CM, Diamantopoulos A, Straub DW, et al. Common Beliefs and Reality About PLS: Comments on Rönkkö and Evermann (2013). Organ Res Methods. 2014 Apr 1;17(2):182–209.

8. Rigdon EE. Choosing PLS path modeling as analytical method in European management research: A realist perspective. Eur Manag J. 2016 Dec;34(6):598–605.

9. Rigdon EE, Sarstedt M, Ringle CM. On Comparing Results from CB-SEM and PLS-SEM: Five Perspectives and Five Recommendations. Mark ZFP. 2017;39(3):4–16.

10. Borsboom D, Mellenbergh GJ, Heerden JV. The theoretical status of latent variables. Psychol Rev. 2003;110(2):203–19.

11. Lord FM, Novick MR. Statistical theories of mental test scores. Charlotte, NC: Information Age Publ; 1968. 568 p. (The Addison-Wesley series in behavioral science: quantitative methods).

12. McDonald RP. Test theory: a unified treatment. Mahwah, N.J: L. Erlbaum Associates; 1999. 485 p.

13. Bollen KA, Bauldry S. Three Cs in Measurement Models: Causal Indicators, Composite Indicators, and Covariates. Psychol Methods. 2011 Sep;16(3):265–84.

14. Embretson SE. The new rules of measurement. Psychol Assess. 1996;341–9.

15. Kline RB. Principles and practice of structural equation modeling. Fourth edition. New York: The Guilford Press; 2016. 534 p. (Methodology in the social sciences).

16. DeVellis R. Scale Development: Theory and Applications [Internet]. 4th ed. Los Angeles: SAGE Publications Ltd; 2017 [cited 2019 Jul 29]. Available from: https://us.sagepub.com/en-us/nam/scale-development/book246123

17. Sijtsma K, Ark LA van der. Measurement models for psychological attributes. Boca Raton London New York: CRC Press; 2021. 407 p. (Chapman & Hall/CRC statistics in the social and behavioral sciences).

18. Cho E. Making Reliability Reliable: A Systematic Approach to Reliability Coefficients. Organ Res Methods. 2016 Oct 1;19(4):651–82.

19. Rindskopf D, Rose T. Some Theory and Applications of Confirmatory Second-Order Factor Analysis. Multivar Behav Res. 1988 Jan;23(1):51–67.

20. Law KS, Wong CS, Mobley WH. Toward a Taxonomy of Multidimensional Constructs. Acad Manage Rev. 1998 Oct;23(4):741.

21. Lee N, Cadogan JW. Problems with formative and higher-order reflective variables. J Bus Res. 2013 Feb 1;66(2):242–7.

22. Chen FF, West S, Sousa K. A Comparison of Bifactor and Second-Order Models of Quality of Life. Multivar Behav Res. 2006 Jun 1;41(2):189–225.

23. Yung YF, Thissen D, McLeod LD. On the relationship between the higher-order factor model and the hierarchical factor model. Psychometrika. 1999 Jun 1;64(2):113–28.

24. Ward JT, Nobles MR, Fox KA. Disentangling Self-Control from Its Elements: A Bifactor Analysis. J Quant Criminol. 2015 Dec;31(4):595–627.

25. Eid M. Multi-Faceted Constructs in Abnormal Psychology: Implications of the Bifactor S - 1 Model for Individual Clinical Assessment. J Abnorm Child Psychol. 2020 Jul;48(7):895–900.

26. Eid M, Geiser C, Koch T, Heene M. Anomalous results in G-factor models: Explanations and alternatives. Psychol Methods. 2017 Sep;22(3):541–62.

27. Holzinger KJ, Swineford F. The Bi-factor method. Psychometrika. 1937 Mar 1;2(1):41–54.

28. Avila ML, Stinson J, Kiss A, Brandão LR, Uleryk E, Feldman BM. A critical review of scoring options for clinical measurement tools. BMC Res Notes. 2015 Oct 28;8:612.

29. McNeish D, Wolf MG. Thinking twice about sum scores. Behav Res Methods. 2020 Dec;52(6):2287–305.

30. Nunnaly J, Bernstein I. Psychometric Theory. New York: McGraw-Hill; 1994.

31. DiStefano C, Zhu M, Mîndrilã D. Understanding and Using Factor Scores: Considerations for the Applied Researcher. Pract Assess Res Eval [Internet]. 2009 [cited 2021 Dec 3];14(20). Available from: https://scholarworks.umass.edu/pare/vol14/iss1/20/

32. Grice JW. Computing and evaluating factor scores. Psychol Methods. 2001 Dec;6(4):430–50.

33. Dana J, Dawes RM. The Superiority of Simple Alternatives to Regression for Social Science Predictions. J Educ Behav Stat. 2004;29(3):317–31.

34. Reise SP, Bonifay WE, Haviland MG. Scoring and Modeling Psychological Measures in the Presence of Multidimensionality. J Pers Assess. 2013 Mar;95(2):129–40.

35. Reise SP, Waller NG, Comrey AL. Factor analysis and scale revision. Psychol Assess. 2000 Sep;12(3):287–97.

36. Reise SP, Moore TM, Haviland MG. Bifactor Models and Rotations: Exploring the Extent to which Multidimensional Data Yield Univocal Scale Scores. J Pers Assess. 2010 Nov;92(6):544–59.

37. Rodriguez A, Reise SP, Haviland MG. Evaluating bifactor models: Calculating and interpreting statistical indices. Psychol Methods. 2016 Jun;21(2):137–50.

38. Bollen KA, Diamantopoulos A. In defense of causal-formative indicators: A minority report. Psychol Methods. 2017 Sep;22(3):581–96.

39. Diamantopoulos A, Siguaw JA. Formative Versus Reflective Indicators in Organizational Measure Development: A Comparison and Empirical Illustration. Br J Manag. 2006;17(4):263–82.

40. Sarstedt M, Hair JF, Ringle CM, Thiele KO, Gudergan SP. Estimation issues with PLS and CBSEM: Where the bias lies! J Bus Res. 2016 Oct 1;69(10):3998–4010.

41. Bollen KA. Structural equations with latent variables. New York: Wiley; 1989. 514 p. (Wiley series in probability and mathematical statistics).

42. Henseler J. Composite-based structural equation modeling: analyzing latent and emergent variables. New York, NY: The Guilford Press; 2021. (Methodology in the social sciences).

43. Schuberth F, Henseler J, Dijkstra TK. Confirmatory Composite Analysis. Front Psychol. 2018;9:2541.

44. Schuberth F, Rademaker ME, Henseler J. Estimating and assessing second-order constructs using PLS-PM: the case of composites of composites. Ind Manag Data Syst. 2020 Sep 1;120(12):2211–41.

45. Cenfetelli, Bassellier. Interpretation of Formative Measurement in Information Systems Research. MIS Q. 2009;33(4):689.

46. Rigdon EE. Rethinking Partial Least Squares Path Modeling: In Praise of Simple Methods. Long Range Plann. 2012 Oct;45(5–6):341–58.

47. Coltman T, Devinney TM, Midgley DF, Venaik S. Formative versus reflective measurement models: Two applications of formative measurement. J Bus Res. 2008 Dec 1;61(12):1250–62.

48. Howell RD, Breivik E, Wilcox JB. Reconsidering formative measurement. Psychol Methods. 2007 Jun;12(2):205–18.

49. Esposito Vinzi V, Chin WW, Henseler J, Wang H, editors. Handbook of Partial Least Squares: Concepts, Methods and Applications [Internet]. Berlin, Heidelberg: Springer Berlin Heidelberg; 2010 [cited 2021 Mar 5]. Available from: http://link.springer.com/10.1007/978-3-540-32827-8

50. Bollen KA. Interpretational confounding is due to misspecification, not to type of indicator: comment on Howell, Breivik, and Wilcox (2007). Psychol Methods. 2007 Jun;12(2):219–28; discussion 238-245.

51. Aguirre-Urreta MI, Rönkkö M, Marakas GM. Omission of Causal Indicators: Consequences and Implications for Measurement. Meas Interdiscip Res Perspect. 2016 Jul 2;14(3):75–97.

52. Edwards JR, Bagozzi RP. On the nature and direction of relationships between constructs and measures. Psychol Methods. 2000;5(2):155–74.

53. Guyon H, Tensaout M. Are Formative Indicators Superfluous? An Extension of Aguirre-Urreta, Rönkkö, and Marakas Analysis. Meas Interdiscip Res Perspect. 2016 Jul 2;14:101–4.

54. Wilcox JB, Howell RD, Breivik E. Questions about formative measurement. J Bus Res. 2008 Dec 1;61(12):1219–28.

55. Bagozzi RP. Measurement and Meaning in Information Systems and Organizational Research: Methodological and Philosophical Foundations. MIS Q. 2011;35(2):261–92.

56. Cadogan JW, Lee N. Improper use of endogenous formative variables. J Bus Res. 2013 Feb 1;66(2):233–41.
